# Siblings’ Influence on the Motor Competence of Preschoolers

**DOI:** 10.3390/children8030204

**Published:** 2021-03-08

**Authors:** Luis P. Rodrigues, Carlos Luz, Rita Cordovil, Rui Mendes, Rita Alexandre, Vítor P. Lopes

**Affiliations:** 1 Escola Superior Desporto e Lazer de Melgaço, Instituto Politécnico de Viana do Castelo, 4900-347 Viana do Castelo, Portugal; 2 Research Center in Sports Sciences, Health and Human Development (CIDESD), 5000-801 Vila Real, Portugal; vplopes@ipb.pt; 3 Escola Superior de Educação de Lisboa, Instituto Politécnico de Lisboa, 1549-003 Lisboa, Portugal; carlosl@eselx.ipl.pt; 4 Centro Interdisciplinar de Estudos Educacionais, 1549-003 Lisboa, Portugal; 5 Faculdade de Motricidade Humana, Universidade de Lisboa, 1499-002 Cruz Quebrada-Dafundo, Portugal; ritacordovil@fmh.ulisboa.pt; 6 Interdisciplinary Center for the Study of Human Performance (CIPER), 1499-002 Cruz Quebrada-Dafundo, Portugal; 7 Escola Superior de Educação, Instituto Politécnico de Coimbra, 3030-329 Coimbra, Portugal; rmendes@esec.pt; 8 Centro de Investigação em Desporto e Atividade Física (CIDAF-UC), 3040-248 Coimbra, Portugal; 9 Department of Physical Education, Colégio Pedro Arrupe, 1990-529 Lisboa, Portugal; ritasra@netcabo.pt; 10 Escola Superior de Educação, Instituto Politécnico de Bragança, 5300-253 Bragança, Portugal

**Keywords:** only child, MCA, early childhood, household, motor performance

## Abstract

The number of single-child families has been rising steadily in recent years, resulting in a childhood absent of sibling relationships. Being an only child has been shown to have a negative impact on physical fitness, somatic fitness, and motor development. In this study, we aimed to understand how living with and without siblings can impact the motor competence of children. One hundred and sixty-one children (87 boys, 74 girls) from 3.0 to 6.0 years of age (34 only children, 125 siblings) and with no known motor or cognitive disability were assessed using the Motor Competence Assessment (MCA). Their standardized results on the three MCA subscales (stability, locomotor, and manipulative) and total MCA were used to group them into high, average, and low motor competence groups. Motor competence percentile distribution of the sibling and only child group condition was compared using chi-square tests. Results showed a significative and positive association between the sibling condition and the distribution between the three MC groups (chi-square = 6.29; *p* = 0.043), showing that children in a household with siblings, independent of their age and sex, show a clear tendency for developing better motor competence.

## 1. Introduction

In recent years, the number of single-child families has been rising steadily. In 2019, in the EU-27, almost half of the households with offspring included only one child (47.4%), and Portugal was the country with the highest rate of single-child families (58.3%) within households with offspring [1]. Different reasons seem to contribute to this trend, such as women having children later in life, the need for the mother to carrying on working due to financial issues, and an increase in divorce rates [2]. Being an only child could have implications on child development. Siblings are an essential component of family systems and play a significant role in development and learning [3,4]. Siblings demonstrate the ability to teach one another during home interactions [3,5] but their age difference implies different experiences in the household. First-born children engage in leadership, teaching, and helping roles, whereas second-born children are more likely to imitate, to follow and be a learner [3,5], having the benefit of learning from an older sibling, leading sometimes to faster development for second-born children [6,7].

Motor competence (MC), defined as a person’s proficiency to execute motor skills as well as the underlying mechanisms including motor coordination and control [8,9,10] is associated with health-related behaviors and attributes such as physical activity (PA) and body mass index (BMI) [11,12].

In 2008, Stodden and colleagues presented a developmental approach proposing motor competence as the primary underlying mechanism that promotes engagement in physical activity and healthy lifestyles [13]. The idea is that there is a relationship between MC and physical activity that starts in early childhood and strengthens over developmental time. According to Stodden’s model, if children attain good motor competence during childhood, exhibiting a proficient level in fundamental movement skills (e.g., running, jumping, throwing, catching, and kicking), they will be able to participate with success in different activities, games and sports [10,13], and this will positively affect their PA participation and weight status for the future. In fact, MC has been shown to be related to different health indicators, being inversely associated with fat mass development in children and adolescents [12,14], and positively associated with health-related fitness [15,16,17], and physical activity [18,19,20].

Children’s development is influenced by a wide variety of biological and environmental factors that interact across time [21]. According to Bronfenbrenner’s ecological model [22,23], the child’s development occurs largely by interactions and relationships between the child and his/her environment, which is conceived as a set of nested structures, namely the microsystem, mesosystem, exosystem, macrosystem and chronosystem. The microsystem is the most influential level of the ecological systems theory and it refers to the immediate context in which face-to-face interactions occur, such as the child’s home or school. The child’s development is facilitated, at a proximal level, by interactions with the persons in his/her microsystems, such as the child’s parents and siblings.

The consequences for motor development of being an only child did not deserve as much attention from early studies as the potential personality differences. However, with the increasing number of single-child families, the motor development of these children became a subject of interest. Studies highlighted that the presence of siblings and peers is related to enriched stimulation for motor development [24,25]. During infancy, older siblings influence the earlier onset of younger siblings’ motor milestones (crawling and walking) [26], and in childhood, having siblings is associated with higher levels of physical activity [27,28], better physical fitness [29], and lower likelihood of being overweight or obese [30,31], probably because children who have brothers or sisters to play with can easily be more active. Specifically, the existence of older siblings has been linked with positive changes in the youngest siblings’ physical activity over time [32]. The positive effect of siblings on physical activity was clear even during the COVID-19 confinement, as reported by children’s parents [33], highlighting that having other children to play with in one’s microsystem was important even when the times (chronosystem) led to a general decline in physical activity.

The effect of siblings on the three dimensions of motor competence (stability, locomotor and manipulative) at an age where fundamental motor skills should emerge and mature (3 to 6 years of age) has not yet been fully addressed in the literature, and thus it is the aim of this study.

## 2. Materials and Methods

One hundred and sixty-one children, 87 boys and 74 girls, from nine preschool classrooms of a private school located in Lisbon, Portugal, were assessed in terms of their motor competence related to a school project. The aim of the project was to improve motor competence in the early ages and participants’ results were used as a convenience sample in this study. Children’s age ranged from 3.0 to 6.0 years, with no known motor or cognitive disability. Thirty-four children (mean age 4.6 ± 0.70) were only child (15 boys; 19 girls) and 125 children (mean age 4.7 ± 0.79) had siblings (71 boys, 54 girls). A two-way ANOVA showed no decimal age differences between sex (*p* = 0.835), sibling groups (*p* = 0.443), or sex within sibling groups (*p* = 0.282). All children’s families had a high or medium high SES. A posterior sample power analysis showed a power of 0.99 for this sample for the use of a chi-square statistics.

Preschool directors gave permission for data collection, parents or tutors of the children gave their informed consent and children gave their verbal assent prior to data collection. Teachers informed the research team about the sociodemographic information of the participants, reporting the number of brothers and sisters and respective age, based on school records. All procedures were carried out in accordance with the 1964 Helsinki declaration and its later amendments, and the Ethics Committee of the Faculty of Human Kinetics, University of Lisbon (CEFMH Approval Number: 26/2013), approved this study.

Motor competence was assessed with the Motor Competence Assessment (MCA) test battery [34] that is composed of three subscales: stability skills (lateral jumps, shifting platforms), locomotor skills (standing long jump, 10 m shuttle run), and manipulative skills (ball kicking velocity, ball throwing velocity). All tests are quantitative (product-oriented) motor tests without a marked developmental (age) ceiling effect, and of feasible execution (for full description see Rodrigues et al., 2019 [34]). Before starting the test, all participants completed a 10 min general and standardized warm-up. Participants performed all the tests in small groups (approximately 5 children for each task). The examiner was blind to the sibling or no sibling condition and was previously trained in administering all tests. The following requirements were used as standard: a) a proficient demonstration of each test technique was provided along with a verbal explanation; b) every participant experimented with each task before the actual test administration; c) the instructions emphasized that participants should try to perform the task at their maximum potential (e.g., “as fast as possible” for the stability tests and 4 × 10 shuttle run; “as far as possible” for the standing long jump; and “as hard as possible” for the manipulative tests); d) motivational feedback was given, but no verbal feedback on skill performance was provided. The MCA testing took place at each preschool gymnasium.

Results on each test of the three subscales were transformed into a percentile value relative to age and sex, according to the MCA norms [34]. Total MCA was calculated by the average of the subscales’ percentiles. *T*-tests we used to compare groups for each MCA subscale and total. Furthermore, children were divided into three motor competence groups (low MC, average MC, and high MC) according to their percentile score on the MCA (total and subscales). Chi-square tests were used to compare the distribution of only children and siblings on the MC group’s classification for total MCA and subscales. Post-hoc tests with Bonferroni adjustment compared values (sibling, no-sibling) for each MCA classification. The Cramer V coefficient was used for determining the strength of the chi-square results (>0.5—high association; 0.3 to 0.5—moderate association; 0.1 to 0.3—low association; 0 to 0.1—little if any association). The IBM^®^ SPSS^®^ v25.0 statistical software was used in the analysis.

## 3. Results

In Table 1, we describe the frequency, mean and standard deviation of each MCA subscale and total of the only child and sibling children’s percentile scores. When analyzing all the children, children with siblings show a higher percentile average for total MCA and all subscales. These differences between the sibling condition groups are nevertheless not statistically significant (all *p* > 0.05).

Analyzing the distribution of the only children and siblings on the proficiency groups (Table 2), we found a significantly different distribution (*p* = 0.043) in the MCA total classification, showing a statistically significant greater percentage of children with siblings in the higher proficiency group (37%) compared with the only child (18%) along with a statistically significant lower percentage of siblings in the average proficiency group (30% vs. 50%), although the strength of the association was low (Cramer’s V = 0.20). No other statistically significant differences were found between distributions for all other MCA subscales, but the within subscales percentage of children with siblings was always higher in the High-MC category (35% vs. 24%, 34% vs. 27%, and 34% vs. 30%, respectively, for the stability, locomotor, and manipulative subscales).

## 4. Discussion

This study aimed to understand whether being an only child may be related to motor competence. Differences between children with and without siblings did not achieve significance when compared using independent-samples *t*-tests. Nevertheless, children who live with siblings show a significantly different and advantageous distribution for the total MC categories (e.g., higher percentage in the high MC categorization). No differences were found when the performances in each subscale were independently analyzed, although a higher percentage of children with siblings was found for all high tertial groups of motor competence on all MCA subscales, when compared with only children.

In general, research showed that having siblings seems to benefit children’s motor performance [24,35,36]. Krombholz [24] tested the motor coordination, physical fitness, and manual dexterity of 3.5- to 7-year-old children and found that children with older sisters or brothers outperformed only or firstborn children, with significant differences in balancing, lateral jump, shuttle run, hopping on the right foot, and arm hanging. Kwon and O’Neill [36] also analyzed the performance of 329 children aged 3–5 years and verified that living with young sibling(s) was associated with better locomotor skills and living with older sibling(s) aged 6–17 years was associated with better object control skills. Nevertheless, the KTK battery [37] used in Krombholz’s study lacks manipulative tasks, while the Test of Gross Motor Development—2nd Edition (TGMD-2) [38] used by Kwon and O’Neill assesses manipulative or object control tasks, but does not assess stability tasks.

Rebelo and collaborators tested infants and children until 4 years of age and the results indicated that the advantage of having siblings started to appear after 2 years of age, but most effect sizes were low [35]. Regarding gross motor skills, differences were mainly in object control skills and locomotion skills. Results for the stability skills did not show a clear tendency since they were significant in the 2-year-old group but not in the 3-year-old group.

Additionally, other studies showed that the presence of another child can facilitate more locomotor-related physical activity (e.g., running) and children were more likely to explore the surrounding environment and objects [5,39]. Children, especially young children, tend to copy behaviors (imitation) of siblings as a normal process of development [5] and have a longer duration of interaction with their siblings than anyone else [40,41], which today may be even more evident due to the risk-aversion culture, which strongly conditions the physical activity of children [42], especially in only or firstborn children [24].

The results of the present study show little or no evidence for differences between only children and children with siblings. However, given that the motor competence of children needs time to consolidate, these differences can be amplified with age (from 6 years old) with increasing time for sharing involvement and experiences together. The fact that such a difference (even small) in total motor competence can be detected at this early age, along with the tendency for children with siblings to have higher average values for all MCA subscales (see Table 1), can be indicative that sharing the household with siblings can be advantageous for motor competence development. We should stress that only the sibling condition (to have or have not siblings) was considered in these results. Because a convenience sample drawn from an intervention project was used in this study, no data on birth order, age difference between siblings or sibling sex was available for the analysis. Even so, as in several other studies, there was a trend showing the advantage of having siblings for motor development. In fact, effects from environmental settings are never straightforward, determined immediately after the initial appearance of the event (e.g., sibling birth), rather we look for hypothetical motor competence differences related to an environmental variable (siblings) during a rapid developmental period in childhood. Motor development and related motor competence of children in the first two to three years of life rely immensely on maturation [43]. Children’s rudimentary movements are shown to emerge even in the absence of stimulation. After that, children’s fundamental motor skills are developed and those are susceptible to stimulation, including the presence of siblings [40,43], but this effect cannot be described by a direct pathway, rather an indirect (mediated or moderated) pathway. The mere presence of a sibling in the house does not ensure that the child will be more motor competent, rather it is the stimulation, the enriched environment, and the increased movement that a sibling can provide that play a role. Children’s motor development is a strongly embodied process, but is certainly affected by environmental factors, and clearly is enabled according to the ecological fit between the child’s own body and environmental and cultural challenges [44]. All these effects have a necessary time demand since they are developmental in nature. Small differences in children’s pathways at these early ages can be amplified through their developmental time into bigger differences. That is why, given the small amount of living time with their brothers or sisters, to expect a very significative effect on motor competence is unreal under a developmental perspective. Differences in motor skill performance are more easily found given the difference between stimulation and motor skill performance, as Rebelo and colleagues [35] found when using the fundamental motor skills assessment. Motor competence must be understood as a latent variable, as a capacity that develops to a lifelong effect, and because of that it is expected to take longer to be established.

This study is not without limitations. A convenience sample was used, and the socioeconomic status of the families was higher than average for Portugal, which could have influenced the children’s MC results. Additionally, potential covariates related to the child, such as children’s weight status and levels of extra-curricular physical activity, were not assessed. Finally, other variables relative to peer and family involvement in physical activities were not accounted for, namely the interaction with peers in school and outside the school, sports participation, or the age difference and sex between siblings. Further studies on the matter should consider all these variables, and if possible, use longitudinal data.

## 5. Conclusions

This study shows that in early age, children in a household with siblings, independent of their age and sex, have a greater chance of being classified in the high and average motor competence groups regarding their total motor competence, when compared to children without siblings. These results can be indicative of a tendency for developing better motor competence in the future. Regardless of the need for further enlightenment on this situation, it is important that families and different agents that work with children consider the possible effects that being an only child can have on the child’s motor development.

## Figures and Tables

**Table 1:** Number, mean and standard deviation of the percentile scores within proficiency groups of children with and without siblings on the MCA and subscales.

	Total MCA			Stability			Locomotor			Manipulative		
	N	M	SD	N	M	SD	N	M	SD	N	M	SD
Low MC												
Only child	11	28.8	9.7	9	16.7	13.7	14	16.5	7.0	11	31.7	12.4
Siblings	42	27.7	8.7	44	21.2	9.2	39	15.8	9.3	42	28.1	10.6
Average MC												
Only child	17	48.7	5.3	17	44.5	8.5	11	45.2	10.1	13	53.9	5.3
Siblings	37	50.4	5.9	37	50.5	6.6	43	47.3	9.0	41	52.8	5.0
High MC												
Only child	6	71.4	6.8	8	73.5	7.2	9	76.4	10.8	10	76.0	10.7
Siblings	46	71.7	9.9	44	77.7	12.2	43	79.6	10.8	42	79.6	10.8
TOTAL												
Only child	34	46.3	16.4	34	43.9	22.5	34	41.6	26.1	34	53.2	20.0
Siblings	125	50.6	20.4	125	49.8	25.7	125	48.6	27.7	125	53.5	23.1

MCA—Motor Competence Assessment; MC—motor competence.

**Table 2:** Number of children and percentage within the category (only child or having siblings) per MC groups of MCA total and subscales.

	MCA Total		Stability		Locomotor		Manipulative	
	Only Child	Siblings	Only Child	Siblings	Only Child	Siblings	Only Child	Siblings
Low MC	11	42	9	44	14	39	11	42
	32%	33%	27%	35%	41%	31%	32%	34%
Average MC	17 *	37 *	17	37	11	43	13	41
	50%	30%	50%	30%	32%	34%	38%	33%
High MC	6 *	46 *	8	44	9	43	10	42
	18%	37%	24%	35%	27%	34%	30%	34%
Chi-Square	6.29		5.00		1.35		0.39	
df	2		2		2		2	
Sig.	0.043		0.082		0.510		0.852	
Cramer’s V	0.20		0.18		0.09		0.05	

MCA—Motor Competence Assessment; MC—motor competence. * Post-hoc significant difference between row cell values, after Bonferroni adjustment.

## Data Availability

The data presented in this study are available on request from the corresponding author. The data are not publicly available due to the fact that not all parents have given proper consent for a public display of data.
